# Effectiveness and safety of reactive focal mass drug administration (rfMDA) using dihydroartemisinin–piperaquine to reduce malaria transmission in the very low-endemic setting of Eswatini: a pragmatic cluster randomised controlled trial

**DOI:** 10.1136/bmjgh-2021-005021

**Published:** 2021-06-30

**Authors:** Sibonakaliso Vilakati, Nontokozo Mngadi, Jade Benjamin-Chung, Nomcebo Dlamini, Mi-Suk Kang Dufour, Brooke Whittemore, Khayelihle Bhangu, Lisa M Prach, Kimberly Baltzell, Nomcebo Nhlabathi, Calisile Malambe, Bongani Dlamini, Danica Helb, Bryan Greenhouse, Gugu Maphalala, Deepa Pindolia, Muhindo Kalungero, Getahun Tesfa, Roly Gosling, Nyasatu Ntshalintshali, Simon Kunene, Michelle S Hsiang

**Affiliations:** 1 National Malaria Program, Ministry of Health, Manzini, Eswatini; 2 Clinton Health Access Initiative, Mbabane, Swaziland; 3 Epidemiology & Biostatistics, University of California, Berkeley, California, USA; 4 Malaria Elimination Initiative, University of California, San Francisco, California, USA; 5 Medicine, University of California, San Francisco, California, USA; 6 Pediatrics, The University of Texas Southwestern Medical Center, Dallas, Texas, USA; 7 Family Health Care Nursing, University of California, San Francisco, California, USA; 8 National Clinical Laboratory Services, Mbabane, Swaziland; 9 Medicine, Good Shepherd Hospital, Siteki, Swaziland; 10 Paediatrics, Raleigh Fitkin Memorial Hospital, Manzini, Swaziland; 11 Pediatrics, University of California, San Francisco, San Francisco, California, USA

**Keywords:** malaria, public health

## Abstract

**Introduction:**

To reduce malaria transmission in very low-endemic settings, screening and treatment near index cases (reactive case detection (RACD)), is widely practised, but the rapid diagnostic tests (RDTs) used miss low-density infections. Reactive focal mass drug administration (rfMDA) may be safe and more effective.

**Methods:**

We conducted a pragmatic cluster randomised controlled trial in Eswatini, a very low-endemic setting. 77 clusters were randomised to rfMDA using dihydroartemisin–piperaquine (DP) or RACD involving RDTs and artemether–lumefantrine. Interventions were delivered by the local programme. An intention-to-treat analysis was used to compare cluster-level cumulative confirmed malaria incidence among clusters with cases. Secondary outcomes included safety and adherence.

**Results:**

From September 2015 to August 2017, 222 index cases from 47 clusters triggered 46 RACD events and 64 rfMDA events. RACD and rfMDA were delivered to 1455 and 1776 individuals, respectively. Index case coverage was 69.5% and 62.4% for RACD and rfMDA, respectively. Adherence to DP was 98.7%. No serious adverse events occurred. For rfMDA versus RACD, cumulative incidences (per 1000 person-years) of all malaria were 2.11 (95% CI 1.73 to 2.59) and 1.97 (95% CI 1.57 to 2.47), respectively; and of locally acquired malaria, they were 1.29 (95% CI 1.00 to 1.67) and 0.97 (95% CI 0.71 to 1.34), respectively. Adjusting for imbalance in baseline incidence, incidence rate ratio for rfMDA versus RACD was 0.93 (95% CI 0.54 to 1.62) for all malaria and 0.84 (95% CI 0.42 to 1.66) for locally acquired malaria. Similar results were obtained in a per-protocol analysis that excluded clusters with <80% index case coverage.

**Conclusion:**

In a very low-endemic, real-world setting, rfMDA using DP was safe, but did not lower incidence compared with RACD, potentially due to insufficient coverage and/or power. To assess impact of interventions in very low-endemic settings, improved coverage, complementary interventions and adaptive ring trial designs may be needed.

**Trial registration number:**

NCT02315690.

**Key questions**

**What is already known?**

- Reactive case detection (RACD), or malaria testing and treatment in the vicinity of passively detected malaria cases, is a standard of care intervention used in low and very low transmission settings aiming for malaria elimination.
- Despite the use of RACD, progress towards malaria elimination has stalled in many countries and new strategies are needed.
- Reactive focal mass drug administration (rfMDA) is a transmission reducing strategy that has been shown to be effective in a low transmission setting, but there are no trial data from a very low transmission setting.

**What are the new findings?**

- In a pragmatic, cluster randomised controlled trial of rfMDA using dihydroartemisinin–piperaquine compared with RACD, we found that rfMDA was safe.
- rfMDA resulted in lower cumulative incidence, but we were unable to confirm its effectiveness compared with RACD, potentially due to imperfect coverage and/or insufficient power.

**Key questions**

**What do the new findings imply?**

- When implemented in a real-world, very low transmission setting, rMDA was safe but evidence regarding its effectiveness to reduce transmission was weak.
- The challenge to show a statistically significant impact of a targeted community-based intervention in a very low transmission setting highlights the potential need for improved coverage, complementary interventions or adaptive ring trial designs.

## Background

Since 2000, many countries have scaled up effective malaria control interventions, resulting in reductions in malaria burden and a renewed goal to eradicate malaria worldwide by 2050.^1^ When the goal is to interrupt transmission, it may be necessary to treat not only symptomatic malaria but also asymptomatic infections which perpetuate ongoing transmission and represent an increasing proportion of all infections in low transmission settings.^2 3^

In countries aiming for malaria elimination, one widely practised strategy to address asymptomatic infections is active case detection in household members and neighbours of symptomatic cases recently reported from health facilities, also known as reactive case detection (RACD).^4–6^ Since malaria infections cluster in space and time,^3^ RACD can target limited resources to areas at highest risk of harbouring infection. In settings with substantial imported malaria cases that may seed local transmission, RACD also serves as a focal outbreak response.^7^ However, the effectiveness of RACD is limited by the low sensitivity of currently available point-of-care diagnostics to detect low-density and non-falciparum infections.^6 8 9^ Molecular testing such as PCR or loop-mediated isothermal amplification (LAMP) improves sensitivity but is not practical given costs, logistical challenges of specimen collection and transport, and turn around time required for laboratory testing and return visits to treat test-positive individuals.^8^ As such, WHO does not recommend RACD as a strategy to reduce or interrupt transmission.^10^

Mass drug administration (MDA), or the treatment all individuals within a specified area with an effective antimalarial irrespective of infection status,^11 12^ may address some of the challenges of RACD. MDA was a component of many malaria elimination programmes in the mid-20th century but fell out of favour due to concerns regarding its effectiveness, sustainability, cost and fear of accelerating drug resistance. More recent evidence suggests that when implemented in areas of low endemicity and in combination with other interventions, MDA has the potential to sustainably interrupt transmission.^11 12^ Maximising coverage and adherence may also help to mitigate risks of drug resistance.^13^ MDA has recently been recommended by the WHO in areas approaching interruption of transmission where there is good access to treatment, effective implementation of vector control and surveillance, and minimal risk of reintroduction of infection.^14^ However, a dearth of definitive evidence on its effectiveness, safety and feasibility remains.^15^

Eswatini (formerly Swaziland) is among 21 countries worldwide that were identified by WHO as the most likely to reach zero indigenous cases by 2020.^16^ However, several of these countries including Eswatini continue to experience persistent local transmission and resurgence. As a malaria elimination-specific strategy, the Eswatini National Malaria Programme (NMP) has implemented RACD since 2009. Prior studies have confirmed that asymptomatic infections cluster around passively detected index cases, with the highest risk within 200 metres of the index case.^6^ However, in Eswatini RACD using rapid diagnostic tests (RDTs) missed two-thirds of infections and 40% of hotspots compared with more sensitive molecular methods.^6^ Due to logistical challenges, attempts to use more sensitive molecular methods to directly inform treatment have been unsuccessful (N. Dlamini, personal communication).

Reactive focal MDA (rfMDA) is an alternative intervention that builds on RACD for targeting high-risk populations residing near index cases. rfMDA entails MDA without testing in household members and neighbours of recent index cases.^17^ Potential advantages of rfMDA over RACD include treatment of cases missed by RDTs as well as prophylactic protection to all individuals who are at a high risk of infection.^14^ A recent trial of rfMDA using artemether–lumefantrine (AL) from a low transmission setting (defined as infection prevalence 1%–10%^18^) with minimal importation in Namibia reported safe administration and rfMDA reduced locally acquired malaria incidence by approximately 50% compared with RACD.^17^ However, there are no trials of rfMDA from very low transmission settings (defined as infection prevalence >0 but<1%^18^) with a high level of importation, which characterises most near-elimination settings. rfMDA may be more appropriate than blanket MDA in low-endemic settings, since it targets populations where malaria has been recently introduced. Also, there are no trials of rfMDA using dihydroartemisinin–piperaquine (DP), which compared with standard artemisinin-combination therapies such as AL, has favourable characteristics for MDA (less frequent dosing and longer period of protection),^14^ but safety concerns about rare QT-interval prolongation leading to arrhythmia and sudden death exist.^19^

Our objective in this trial was to evaluate the effectiveness of rfMDA using DP, compared with RACD, for reducing malaria transmission in the very low transmission setting of Eswatini. Both the rfMDA and RACD interventions were embedded within the Eswatini NMP; as such, this pragmatic trial assessed real-world effectiveness of these interventions when delivered within an existing surveillance and response programme.

## Methods

### Study design and participants

We conducted a pragmatic open-label, cluster randomised controlled trial^20^ between September 2015 and August 2017 in the Kingdom of Eswatini, a low middle-income country in southern Africa. Approximately 30% of the population lives in the eastern malaria-endemic area, which borders Mozambique. *Plasmodium falciparum* is responsible for over 99% of malaria cases. Malaria transmission is unstable and occurs mainly between October and May.^6^ In the transmission year prior to the trial (July 2014–June 2015), 50% of cases were classified as imported.^21^

After major declines in malaria transmission from annual parasite incidence (API) of 3.9 to 0.07 per 1000 population from 1999 to 2009, the NMP reoriented its strategy from control to elimination. Since implementation of the elimination programme and until just prior to this trial, API has remained <1 per 1000 population.

**Figure 1:**
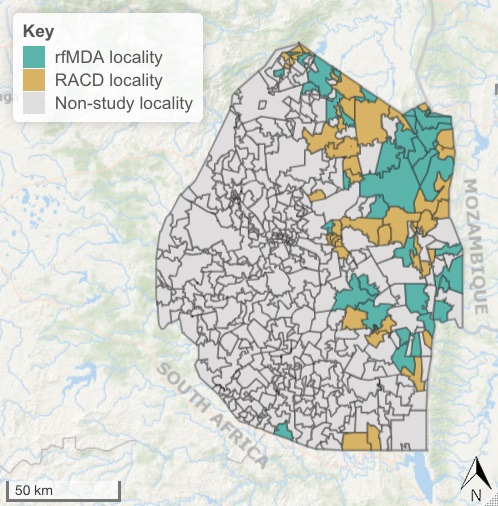
Map of the study area. RACD, reactive case detection; rfMDA, reactive focal mass drug administration.

This pragmatic cluster randomised controlled trial was designed to compare rfMDA and RACD effectiveness as implemented by the Eswatini NMP and in the context of other ongoing interventions including case management, vector control, surveillance and information, communication and education. All 77 of Eswatini’s malaria endemic localities (administrative units), with a total of 209 085 individuals residing in 431 enumeration areas,^22^ were eligible for inclusion. Of the 77 localities, 63 had malaria cases in the 3 years prior to the trial; the remainder did not have cases but had prior historical risk of malaria transmission. We randomised localities with a 1:1 allocation ratio to receive RACD or rfMDA (figure 1), and refer to them as clusters.

During the study, suspected malaria cases presenting to any of Eswatini’s 287 public or private health facilities received malaria testing by RDT or microscopy. Laboratory confirmed cases were reported through the mandatory and toll-free immediate disease notification system. An NMP surveillance team attempted to visit the index case at their home within 48 hours to record household geocoordinates and collect demographic and epidemiological information including an 8-week travel history to classify case origin (ie, local, imported or unknown).

If an index case resided within an RACD or rfMDA study cluster, irrespective of the case origin, their household and neighbouring households were eligible to receive RACD or rfMDA, respectively. Other inclusion and exclusion criteria for triggering the study interventions are shown in online supplemental appendix 1. Briefly, per standard NMCP practice, the ‘target population’ for RACD was households within a 500 m radius of the index case if RACD was not already conducted in the prior 5 weeks. The ‘target population’ for rfMDA was households within a 200 m radius of the index case, but extending beyond 200 m to reach a minimum of 30 individuals. This ‘target population’ was chosen because prior studies showed that the majority of infections near an index case could be captured within this target population and NMP wanted to limited drug administration to those at highest risk.^6^ Following the manufacturer’s recommendation that DP not be repeated within 8 weeks, nor taken more than twice in a year, rfMDA was not repeated if these criteria were met. Other exclusion criteria for DP included: age <9 months; weight <7 kg; pregnancy and breastfeeding, allergy to DP, acute illness including severe malaria, underlying kidney or hepatic problems, personal or family history of QT prolongation, or recent treatment with QT-prolongating medications.

10.1136/bmjgh-2021-005021.supp1 Supplementary data

### Randomisation and masking

To ensure that the baseline risk of malaria was balanced between intervention arms, we used block-stratified randomisation. We assigned the 77 clusters to randomisation blocks by separating them into three risk groups based on incidence in the 3 years prior to the trial and prior historical risk according to NMP. We further stratified each block by whether the size of the population at risk was above or below 650 individuals, resulting in six total strata. A statistician at UC San Francisco (Mi-Suk Kang Dufour) generated the random allocation sequence using SAS (V.9.4M2) to randomly assign 0 or 1 to each cluster within each block and stratum, and the NMP flipped a coin to determine which intervention corresponded to 0 and 1. The intervention delivery team and study investigators were not blinded to intervention assignment due to the nature of interventions.

### Procedures

Households residing in endemic areas received annual indoor residual spraying (IRS) per NMP’s standard approach of targeting high risk areas; cluster-level coverage of IRS was not measured. Households within proximity to index cases received RACD or rfMDA based on the above mentioned inclusion and exclusion criteria.

For RACD, consistent with NMP standard practices, consenting individuals received RDT testing with *P. falciparum*-specific First Response (Premier Medical, Mumbai, India), and a dried blood spot (DBS) was collected for subsequent molecular testing. Per NMP standard practice, RDT-positive individuals were transported to the nearest health facility for treatment with AL (Coartem, Novartis Pharmaceuticals, Kempton Park, South Africa) (As RACD teams typically do not include nurses nor physicians, they are not authorised to administer antimalarials in Eswatini). The study aimed to deliver interventions within 7 days of index case presentation, but allowed up to 5 weeks.

For rfMDA, individuals were targeted for presumptive drug administration using DP (Eurartesim, Sigma Tau, Italy). Field staff assessed whether it was safe to administer DP to enrolled eligible individuals. Individuals ineligible to receive DP were screened using RDTs and transported to the nearest health facility for treatment if they tested positive, and a DBS was collected for subsequent molecular testing. Eligible individuals received the first dose of DP under directly observed therapy and doses for day 2 and day 3 for self-administration. Participants were instructed to go to the nearest health facility if they experienced any illness after taking DP, and they were instructed to contact an on-call study nurse that was available at all hours. The protocol specified pill counts 7–10 days after enrolment in a subsample, but NMP elected to conduct this adherence assessment in all rfMDA participants.

In both arms, demographic and epidemiological information including coverage of vector control interventions was collected. Field staff returned a second and third day to recruit individuals who were initially absent. The study aimed to achieve at least 80% intervention coverage of index cases and 80% coverage of the target population. All study participants were instructed to notify study nurses who were available by telephone 24 hours/day, 7 days/week about any adverse events. Active pharmacovigilance was also conducted during the follow-up visits to assess adherence.

### Laboratory methods

RDT testing was performed using the First Response *P. falciparum* HRP-2 Detection Test (Premier Medical). Molecular assays were conducted at the Swaziland Laboratory Health Services laboratory. DNA was Chelex extracted from DBS and first used for genus-specific LAMP testing. If a sample was positive, *P. falciparum*-specific LAMP testing was then performed (Loopamp Malaria Pan and Pf Detection Kits, Eiken Chemical).^6^ LAMP results were used for research purposes only.

### Outcomes

The primary outcome of the trial was the cluster-level cumulative incidence of all passively detected malaria cases per person years at risk over 2 years of follow-up. Secondary outcomes include safety and adherence (acceptability has been reported elsewhere^23^). Infection prevalence and seroprevalence were originally also secondary outcomes but the endline cross-sectional survey was not conducted due to a shift in priorities within the Ministry of Health.

### Statistical analysis

Based on surveillance data from 2012 to 2015 in areas where RACD was conducted, we assumed an annual incidence of 4 per 1000 individuals, coefficient of variation of 0.9, and type I error of 0.05. Assuming 80% statistical power and a minimum 50% reduction between arms,^6 24^ and with the harmonic mean cluster size being 656, the required number of clusters with at least one index case was 51.^25^

The cumulative incidence in each cluster was calculated as the number of passively detected malaria cases divided by the product of population and follow-up time in each cluster, starting on the date of first index case detection. The first index case in each cluster was excluded from incidence calculations since interventions were delivered after initial index case detection in each cluster.

To estimate intervention effects, we used an intention-to-treat (ITT) approach that excluded clusters with no incident cases during the study period since these clusters did not receive interventions.^26^ The primary analysis was a cluster-level analysis using negative binomial regression models with an offset for cluster population size to estimate incidence rate ratios in each cluster over the study period. The study protocol pre-specified adjusted analyses if there was evidence of baseline imbalance between arms but did not list specific adjustment covariates. Models adjusted for baseline covariates that were associated with the outcome using a likelihood ratio test (p<0.2) and that had a Pearson correlation coefficient with the outcome ≥0.3.^27^ Baseline covariates included: incidence of all cases (2014–2015), incidence of local cases (2014–2015), proportion of imported cases, proportion of houses receiving IRS in the past year, monthly average enhanced vegetation index, monthly average rainfall, monthly average land surface temperature and elevation.

In a secondary analysis, malaria-free survival was compared, and the assumption of proportional hazards was assessed using Schoenfeld residuals testing.^28^ We also performed a per-protocol analysis that excluded clusters in which fewer than 80% of interventions delivered were consistent with intervention assignment; this analysis was not prespecified.

To assess potential contamination due to a lack of buffer zones between clusters, we identified all clusters with contiguous neighbouring clusters and plotted the incidence in each cluster against incidence in the neighbouring cluster. If contamination occurred, and, for example, the rfMDA intervention was highly effective, RACD clusters contiguous to rfMDA clusters with low incidence may have had lower incidence than other RACD clusters. In the RACD arm, there were 9 clusters neighbouring RACD clusters and 8 neighbouring rfMDA clusters; in the rfMDA arm, there were 18 clusters neighbouring rfMDA clusters, and 13 neighbouring RACD clusters. The small number of contiguous neighbouring clusters precluded the use of statistical models to assess whether cluster incidence was associated with the incidence in neighbouring clusters.

## Results

Thirty-eight localities were randomly assigned to receive the RACD intervention, and 39 localities were randomly assigned to receive the rfMDA intervention. There was imbalance in baseline transmission intensity. Cumulative incidence of all malaria in the 3 years preceding the trial (September 2012–August 2015) was higher in the rfMDA arm compared with the RACD arm (6.50 vs 4.19 per 1000, respectively) with a similar trend seen for local cases only, and for all cases and local cases only in the 14 months preceding the trial (table 1). The percentage of cases classified as imported in each cluster in the years prior to the trial was higher in the RACD arm compared with the rfMDA arm (35.8% vs 28.5% for 2012–2015, and 48.0% vs 34.8% for 2014–2015). Mean population size and ecological factors including rainfall, enhanced vegetative index, elevation and daytime land surface temperature were balanced between arms at baseline (table 1).

**Table 1:** Baseline characteristics of clusters included in the trial

Cluster-level characteristic	Overall n=77	RACD n=38	rfMDA n=39
Transmission intensity and control measures, mean (95% CI)			
September 2012–August 2015 (3 years preceding study)			
Cumulative incidence of all cases	5.36 (3.80 to 6.92)	4.19 (2.93 to 5.45)	6.50 (3.65 to 9.35)
Cumulative incidence of local cases	4.03 (3.22 to 4.84)	3.33 (2.17 to 4.49)	4.70 (3.57 to 5.83)
Proportion of cases classified as imported*	32.1 (25.2 to 38.9)	35.8 (24.4 to 47.2)	28.5 (20.4 to 36.6)
July 2014–August 2015 (14 months preceding study†)			
Cumulative incidence of all cases	3.19 (2.22 to 4.16)	2.63 (1.39 to 3.86)	3.73 (2.21 to 5.26)
Cumulative incidence of local cases	2.45 (1.57 to 3.33)	1.81 (0.62 to 3.00)	3.07 (1.75 to 4.39)
Proportion of cases classified as imported‡	41.0 (30.1 to 51.9)	48.0 (30.5 to 65.5)	34.8 (20.7 to 48.9)
Population characteristics, mean (95% CI)			
Size	2715 (2275 to 3156)	2752 (2086 to 3418)	2680 (2070 to 3289)
Ecological factors, median (range)			
Rainfall, mm§	65.9 (36.9 to 92.6)	64.8 (39.6 to 92.6)	66.7 (36.9 to 89.0)
EVI§	0.29 (0.19 to 0.44)	0.28 (0.21 to 0.39)	0.29 (0.19 to 0.44)
Elevation, m	368 (147 to 852)	377 (170 to 589)	355 (147 to 852)
Daytime LST, °C§	31.2 (28.3 to 35.7)	31.4 (28.4 to 35.2)	31.1 (28.3 to 35.7)

Incidences are cases per 1000 population.

*Sample size (n) for overall, RACD and rfMDA were 74, 36 and 38 clusters, respectively.

†Because the National Malaria Programme’s annual reports extend from July to June, this period is included in the baseline data for the 14 months preceding the trial.

‡Sample size (n) for overall, RACD and rfMDA were 52, 25 and 27 clusters, respectively.

§Mean monthly values September 2015–August 2017.

EVI, Enhanced Vegetative Index; LST, land surface temperature; RACD, reactive case detection; rfMDA, reactive focal mass drug administration.

**Figure 2:**
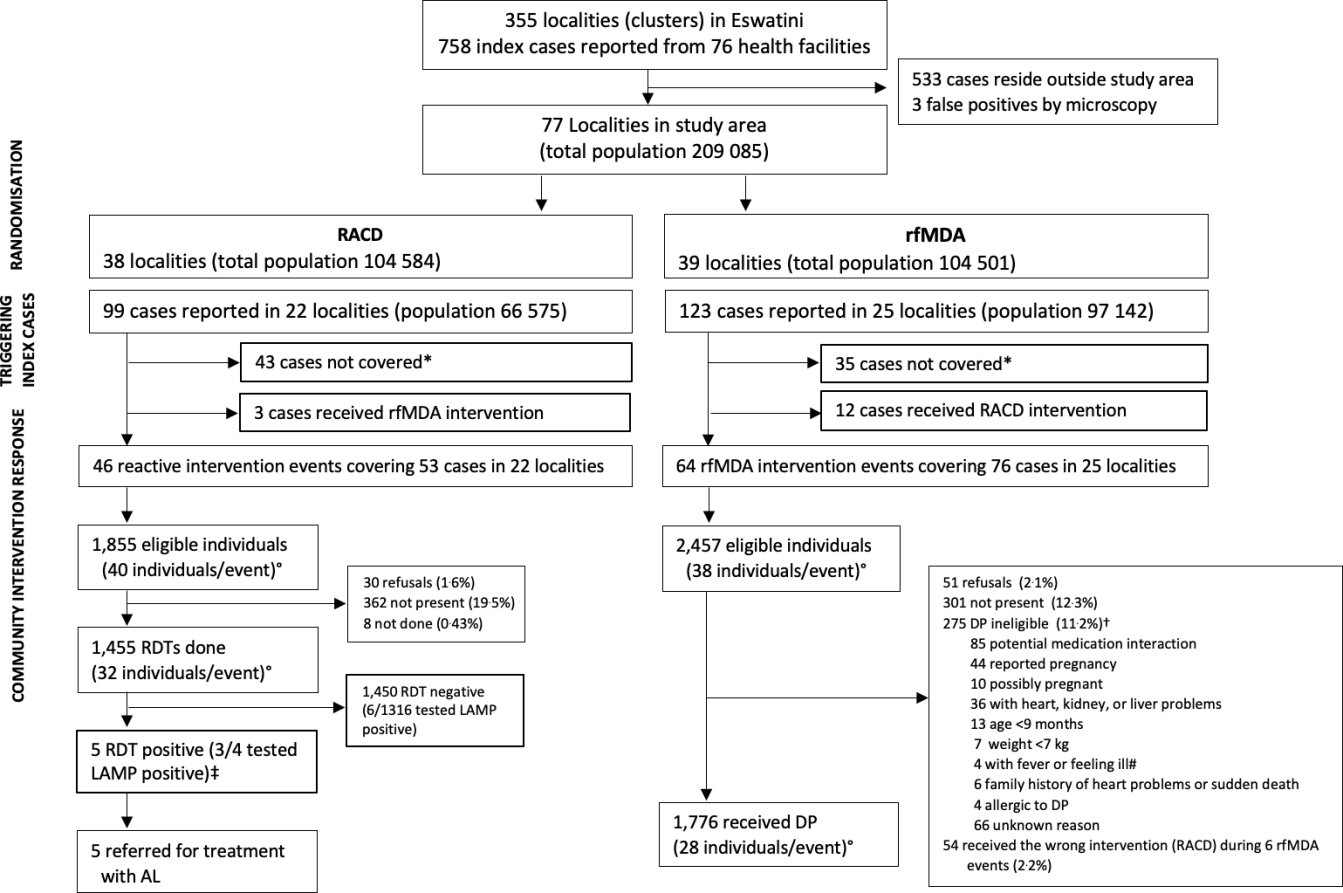
Trial profile showing randomisation and enrolment. *Not covered due to staff limitations, fuel shortages, weather conditions complicating transport, or no study participants being present. °Average per event. †RDT testing conducted in 227 of DP ineligibles (82.5%). As none tested positive, none were referred for treatment with AL. ‡LAMP result not available in 1/5 RDT positives. #Referred to health facilities. No symptomatic malaria cases were found. AL, artemether–lumefantrine; DP, dihydroartemisin-piperaquine; LAMP, loop-mediated isothermal amplification; RACD, reactive case detection; RDT, rapid diagnostic test; rfMDA, reactive focal mass drug administration.

Between September 2015 and August 2017, 110 intervention events reaching 3231 individuals across 39 clusters were implemented during the study period (figure 2). In the RACD arm, 22 of 38 clusters had 99 reported cases; 53 (54%) eligible index cases received RACD, 43 (43%) did not receive interventions, and 3 (3%) incorrectly received rfMDA (table 2). In the rfMDA arm, 25 of 39 clusters had 123 reported cases; 76 (62%) of eligible index cases received rfMDA, 35 (28%) did not receive interventions, and 12 (10%) incorrectly received RACD. Reasons that the target population around index cases did not receive interventions included staff limitations, fuel shortages, weather conditions complicating transport, or no study participants were present. The reasons for implementation of rMDA in a RACD cluster or RACD in a rfMDA cluster (ie, protocol deviation) were not recorded but may have been due to challenges with capturing accurate geocoordinates. The median number of days between index case report and intervention response was 7 (range: 2–27) in the RACD arm and 11 (range: 3–40) in the rfMDA arm. In the rfMDA arm, one cluster had a response time of 40 days; excluding that cluster, the range was 3–26.

**Table 2:** Intervention coverage and response time

	Overall n=47*	RACD n=22*	rfMDA n=25*	P value
Index case coverage†, mean % (95% CI)	65.7 (55.5 to 75.9)	69.5 (53.0 to 86.0)	62.4 (48.8 to 76.0)	0.49
Target population coverage‡, mean % (95% CI)	75.4 (67.2 to 83.6)	70.3 (55.8 to 84.8)	80.2 (71.2 to 89.2)	0.22
Response time, median (range)				
Days between index case report and intervention response	8 (2–40)	7 (2–27)	11 (3–40)	0.09

*Sample size (n) for overall, RACD and rfMDA were 39, 19 and 20 clusters, respectively, for target population coverage and response time.

†Index case coverage was defined as the percentage of eligible index cases that received an intervention averaged across study arm clusters.

‡Target population coverage was defined as the percentage of the target population within 200 m zones around each index case that received an intervention averaged across study arm clusters.

RACD, reactive case detection; rfMDA, reactive focal mass drug administration.

Of the 1855 individuals eligible to receive RACD, 1455 (78.4%) were tested by RDTs. Five RDT-positive secondary cases, of which three were LAMP positive, were referred for treatment with AL. The most common reason for non-receipt of RACD was not present (n=362, 19.5%); only 1.6% (n=30) refused. Of the 2457 individuals eligible to receive rfMDA, 1776 (72.3%) received DP. The most common reasons for non-receipt of rfMDA were not present (n=301, 12.3%) and ineligibility to receive DP (n=275, 11.2%) mainly due to reported potential for medication interaction. Fifty-one (2.1%) of eligible individuals refused to participate. Fifty-four individuals in six rfMDA intervention events received the wrong intervention (RACD). Data on medication type were incomplete as nurses reported sensitivities around participants disclosing use of antiretrovirals (ARVs). No RDT nor LAMP-positive individuals were identified among rfMDA ineligibles. Index cases in rfMDA and RACD arms had a similar distribution of age, sex, case origin (eg, local, imported, or unknown), occupation and bed net ownership between study arms. The percentage of index cases that reported having had their home sprayed in the past year was higher in rfMDA clusters than RACD clusters (28.6% vs 5.3%). For the target population receiving study interventions, there was a similar distribution of age, occupation and vector control coverage. In all study clusters, an average of 35.7% of index cases and 2.8% of the target population reported international travel in the prior 8 weeks during the study period, reflecting high levels of malaria importation in the study site (online supplemental appendix 2).

**Figure 3:**
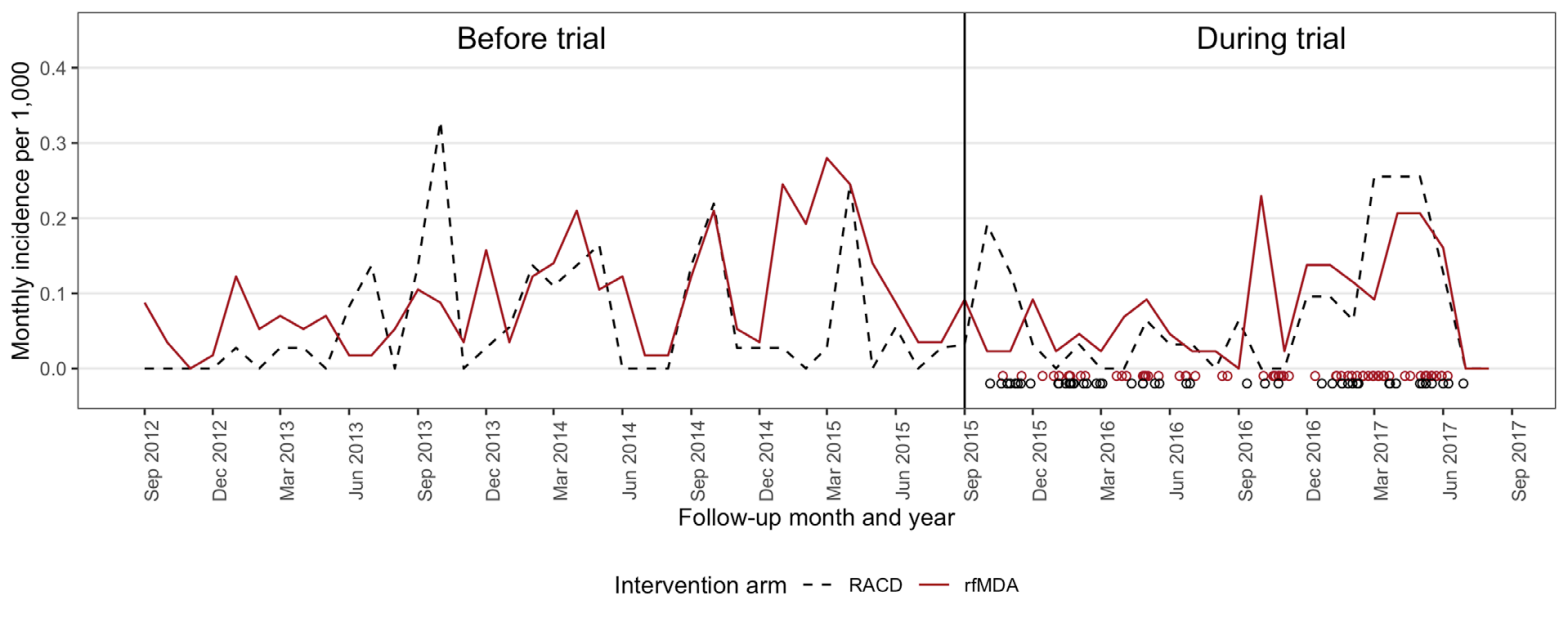
Monthly incidence in each study arm prior to and during the intervention period. Black and red circles show the timing of separate RACD or rfMDA intervention events, respectively. RACD, reactive case detection; rfMDA, reactive focal mass drug administration.

During the follow-up, the average cluster-level cumulative incidence decreased from baseline levels in both the RACD and rfMDA arms, with rfMDA having fewer cases during the final months (February to May) of the second transmission season (figure 3, online supplemental appendix 3). The cumulative incidence from 2015 to 2017 was 2.11 per 1000 in the rfMDA arm compared with 1.97 in the RACD arm (table 3) (total N=47 clusters). In the ITT analysis, crude and adjusted incidence rate ratios (IRRs) were 1.01 (95% CI 0.58 to 1.73) and 0.93 (95% CI 0.54 to 1.62), respectively (table 3). Restricting to local cases only, the adjusted IRR was 0.84 (95% CI 0.42 to 1.66). The per-protocol analysis produced very similar results to the ITT analysis (online supplemental appendix 4).

**Table 3:** Adjusted incidence rate ratios (IRRs) in 2015–2017 comparing clusters assigned to RACD versus rfMDA

Study arm	N clusters	Incidence (cases per 1000 person-years)	Crude IRR	P value	Adjusted IRR*	P value
All cases						
RACD	22	1.97 (1.57 to 2.47)	1 (Ref)	0.99	1 (Ref)	0.81
rfMDA	25	2.11 (1.73 to 2.59)	1.01 (0.58 to 1.73)		0.93 (0.54 to 1.62)	
Local cases only						
RACD	22	0.97 (0.71 to 1.34)	1 (Ref)	0.85	1 (Ref)	0.61
rfMDA	25	1.29 (1.00 to 1.67)	1.06 (0.57 to 1.98)		0.84 (0.42 to 1.66)	

95% CIs for incidence were estimated using the Wilson method. IRRs compared cluster-level incidence in the rfMDA arm to the RACD arm using an intention-to-treat approach and negative binomial models.

*Models adjusted for the following baseline covariates that were associated with the outcome: incidence of all cases from July 2014 to August 2015 (all cases model), incidence of local cases from July 2014–August 2015 (local cases only model).

RACD, reactive case detection; rfMDA, reactive focal mass drug administration

**Figure 4:**
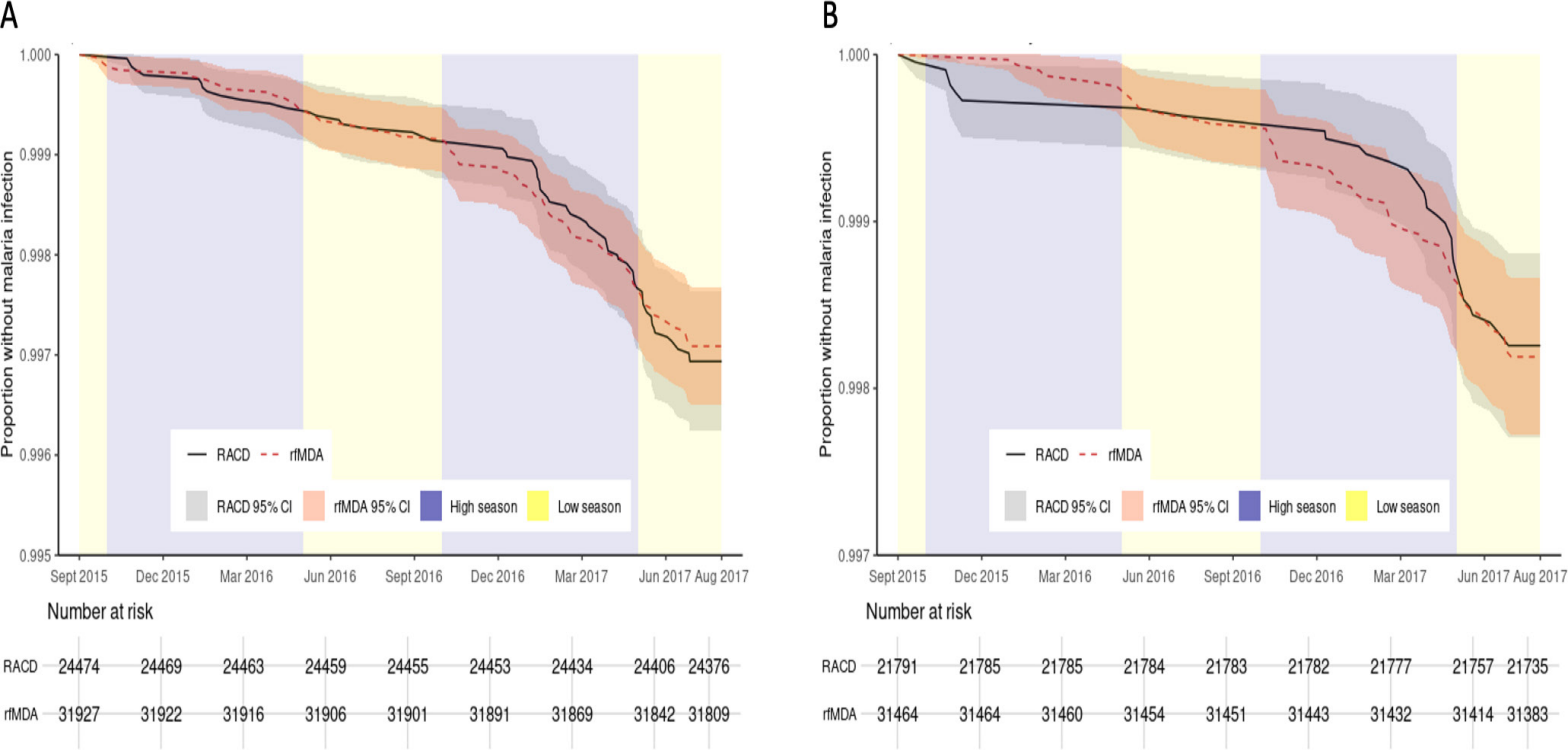
Malaria-free survival curves for the outcomes of (A) all incident malaria cases and (B) local incident malaria cases. High transmission seasons occurred October to may each year. RACD, reactive case detection; rfMDA, reactive focal mass drug administration.

The cumulative proportion of individuals who were not malaria index cases was similar between arms, and 95% CIs overlapped substantially (figure 4A). Restricting to local cases only, the cumulative proportion who were not malaria index cases was higher in the rfMDA arm until approximately 9 months after study initiation (June 2016), and subsequently it was higher in the RACD arm throughout the second high transmission season (October 2016–May 2017); however, at all time points, 95% CIs overlapped (figure 4B).

When comparing incidence in each cluster to incidence in contiguous neighbouring clusters, there was no evident pattern of association between cluster-level incidence and incidence in contiguous neighbouring clusters, suggesting that the risk of contamination in this trial was minimal (online supplemental appendix 5).

Pill counts were conducted in 1114 rfMDA participants (62.7%) and there was complete adherence to the 3-day DP regimen in 1099 (98.7%). Adverse events were experienced by 68 individuals in the rfMDA arm (49 in year 1, 19 in year 2). Based on the WHO severity scale, 54 (79.4%) events were mild and 14 (20.6%) were moderate. The most common complaints were headache, nausea/vomiting and abdominal pain. Of five individuals with adverse events who did not complete the course of DP, all recovered. One had difficulty breathing and chest tightness that could be consistent with DP-associated arrhythmia but the accompanying diarrhoea is less consistent (online supplemental appendices 6 and 7). During the study period, there was only one recorded malaria death in the study area. The infection was locally acquired and the patient lived in an rfMDA cluster, though rfMDA had not previously been conducted in the target area. No AEs were reported in the RACD arm.

## Discussion

In this pragmatic, cluster randomised trial conducted in a very low transmission malaria elimination setting, rfMDA clusters had lower locally acquired malaria incidence compared with RACD clusters, particularly during the second high transmission season, but overall, evidence was weak. Intervention coverage was lower than expected, and malaria occurred in fewer clusters than assumed in the sample size calculation. Adherence to presumptive treatment with DP was high, and as reported elsewhere, acceptability was high.^23^ There were no serious adverse events (SAEs).

Progress towards the 2030 elimination goal in southern Africa has slowed despite coordinated regional efforts and delivery of standard interventions, including preseason IRS, symptomatic case management and RACD.^16^ While RACD aims in part to address asymptomatic reservoirs of transmission, RDTs used in low transmission settings have poor sensitivity and miss many low-density infections.^8^ While blanket MDA would reach all asymptomatic infections, it is logistically difficult to implement at scale, and inefficient in populations with few, highly clustered infections. Potential benefit of blanket MDA may not outweigh risks, leading to low acceptability.^12^

A few trials have evaluated focal MDA delivered to hotspots at the village or subvillage level in low transmission settings and results are inconclusive.^29–32^ In Zanzibar, which most resembles our site due to high coverage of standard interventions, very low transmission intensity, and high rates of importation, negative findings of focal MDA effectiveness were hypothesised to be related to suboptimal timing and the number of MDA rounds, and reintroduction of malaria through importation.^31^ The reactive approach employed in our trial sought to address these issues by targeting the focal MDA to a time and place when transmission risk was highest (eg, where there were recent imported or local cases).

This trial is among three registered trials that evaluated rfMDA in southern Africa. Results from a low transmission setting in Zambia are forthcoming.^33^ A trial in a low transmission setting in Namibia evaluated rfMDA alone and in combination with reactive vector control in comparison to RACD.^17^ Compared with RACD, rfMDA reduced local malaria incidence by 48%, and rfMDA with additional reactive vector control reduced incidence by 74%. There are several key differences between the Namibia trial and this trial. First, the Namibia trial had a higher baseline annual malaria incidence (30 per 1000 compared with 3 per 1000 in this trial) and a lower proportion of imported malaria (2.4% compared with 35.7% in this trial), both of which may facilitate higher impact of focal MDA.^29^ Second, the Namibia trial was largely implemented by a research team, while the Eswatini trial was pragmatic and largely implemented by the local malaria control programme. Coverage in the Namibia trial was also higher compared with this trial (study area index case coverage was >84% compared with 58% overall in this trial). Finally, there was an additive effect for the combination of reactive focal IRS with rfMDA, suggesting that complementary interventions could be considered to improve the impact of rfMDA.

This trial faced several challenges unique to very low incidence settings including strong spatiotemporal clustering and imported malaria.^34^ The number of clusters with at least one index case during follow-up was lower than expected (we expected 51 but observed 47). Thus, the trial was not powered to detect the hypothesised incidence reduction of ≥50%, and null results may reflect a type II error. Second, though the study was cluster randomised, baseline malaria incidence and the percentage of imported cases was higher in the rfMDA arm than the RACD arm. Though analyses adjusted for these factors, it remains possible that unmeasured factors affecting malaria transmission differed between arms. Of note, rfMDA compared with RACD index cases were more likely to have received IRS and this may have been in response to higher transmission in rfMDA arms. Covariate constrained randomisation may have been more effective at achieving baseline balance than stratified randomisation.^35^ When outcomes are rare and clustered, trials require very large cluster numbers to have sufficient statistical power and baseline balance.^36 37^ Future trials of infectious disease interventions in very low transmission settings with strong spatiotemporal clustering may benefit from ring designs, which randomise the group of individuals in proximity to index cases at the time of index case detection rather than randomising fixed geographical areas at baseline.^38^ Adaptive designs^36 37^ that adjust ring trial features such as the number of rings enrolled and the allocation ratio may also increase statistical power in these settings, but to date, only one such trial has been performed.^38^ Finally, including serological endpoints may also increase statistical power.^39^

Implementation factors may have influenced effect estimates. First, index case coverage was lower than the trial’s goal of 80% and was imbalanced across arms (69.5% for RACD and 62.4% for rfMDA). Limitations related to staffing, transport and participant absence contributed to imperfect coverage. Notably, these limitations reflect real-world settings, and imperfect coverage may be similar or greater if rfMDA is implemented outside of a trial setting. In addition, adherence was imperfect and imbalanced across arms: 3% of RACD index cases received rfMDA intervention, and 10% of rfMDA index cases received the RACD intervention. Imperfect coverage and adherence may have diluted the effect of the rfMDA intervention. However, our per-protocol analysis produced similar results to the ITT analysis. Of note, 11% of the rfMDA target population was ineligible to receive DP; the most common reason being potential medication interaction with ARVs, which may have been underreported due to sensitivity around ARV use. Although saquinavir, the only ARV contraindicated for use with DP, is not available in Eswatini, nurses expressed concern that adverse events could be interpreted by the participant as due to ARV, and thus compromise ARV adherence. Where ARV use is common, such as Eswatini which has the highest worldwide incidence of HIV,^40^ better strategies to address safety concerns regarding drug–drug interaction will be needed. Expanding eligibility criteria to include pregnant women, young children and individuals with certain morbidities without contraindicating treatments as has been safely practised by others^41^ could also improve coverage. However, coverage was not associated with incidence, suggesting that differences in coverage between arms were unlikely to affect study findings. Second, intervention response time was substantially higher for two rfMDA clusters compared with the RACD arm. It is possible that there was greater malaria transmission between index case detection and intervention delivery in the rfMDA arm than the RACD arm. Third, study clusters were not separated by geographic buffer zones to minimise contamination, which can occur due to vectors or human movement. However, cluster-level incidence was not correlated between contiguous clusters, suggesting that the chance of contamination in this trial was low.

Importantly, our study is the first to show the safety of rfMDA using DP. The Namibia trial used AL and in comparison, DP may be preferable for MDA due to ease of use and longer protective period (once vs twice daily, and 4–6 weeks vs a few days). Rarely, DP-associated QT-interval prolongation may lead to sudden death (1/~200 000), and in very low-endemic settings the risk-to-benefit ratio may not favour DP.^19^ Here, one participant had symptoms that could be consistent with arrhythmia, and treatment was stopped. Active pharmacovigilance provided by nurses during follow-up visits and their on-call availability likely helped to prevent SAEs.

## Conclusions

This study is the first trial to compare rfMDA and RACD in a very low malaria-endemic setting. As interventions were embedded within an existing NMP, it provides such evidence in realistic implementation conditions. We found that rfMDA was safe. We did not find that incidence was lower in rfMDA vs RACD clusters, but the trial may have had insufficient statistical power and/or coverage to detect a difference in this setting. For rfMDA to be more effective than RACD, improved coverage and/or the addition of complementary interventions may be needed. Ring design, adaptive trials may also increase statistical power of trials in very low transmission settings. Such evidence will be critical to guide countries in their quest to move from very low to no transmission.

## Data Availability

The data that support the findings of this study are not publicly available. Data are available from the authors on reasonable request and with permission of Eswatini Ministry of Health.
