# Supplementary material for: Effectiveness and safety of reactive focal mass drug administration (rfMDA) using dihydroartemisinin–piperaquine to reduce malaria transmission in the very low-endemic setting of Eswatini: a pragmatic cluster randomised controlled trial
Source: BMJ Glob Health. 2021 Jun 30;6(6):e005021. doi: 10.1136/bmjgh-2021-005021 (PMC8246301; doi:10.1136/bmjgh-2021-005021)
Supplement: Supplementary data [file bmjgh-2021-005021supp001.pdf]

Supplementary Appendices

Supplement to: Effectiveness and safety of reactive focal mass drug administration (rfMDA) using dihydroartemisinin-piperaquine to reduce malaria transmission in the very low-endemic setting of Eswatini: a pragmatic cluster randomised controlled trial

Table of Contents

Appendix 1. Inclusion and exclusion criteria 2

Appendix 2. Characteristics of index cases and target population, mean % across clusters (95% CI) during two-year follow-up 3

Appendix 3. Cluster-level incidence in the year prior to the trial (A) and during the trial (B) by intervention arm 5

Appendix 4. Per-protocol analysis: Adjusted incidence rate ratios (IRRs) in 2015–2017 comparing clusters assigned to RACD versus rfMDA 6

Appendix 5. Cluster-level incidence in contiguous clusters by arm 7

Appendix 6. Symptomology of reported adverse events 8

Appendix 7. Symptomology in participants with reported adverse events that did not complete the entire course of dihydroartemisinin-piperaquine (n=5) 9

Appendix 1. Inclusion and exclusion criteria

	Inclusion criteria	Exclusion criteria
Index case (as trigger for RACD or rfMDA in their household members and neighbours)	• Laboratory-confirmed malaria case (either locally transmitted or imported) detected at and reported from a health facility, and<br>• Resided in a study cluster.	• Malaria infection identified through RACD or other active case detection.
RACD intervention	• Non-index case, and<br>• Resided or spent at least one night in the Target Area in the past 5 weeks.	• Target Area overlaps with prior Target Area that received the RACD intervention within the past 5 weeks.
rfMDA intervention	• Non-index case, and<br>• Resided or spent at least one night in the Target Area in the past 5 weeks.	• Target Area overlaps with prior Target Area that received the rfMDA intervention within the past 8 weeks, and<br>• For dihydroartemisinin-piperaquine (DP) specifically (though still eligible for interview):<br>— Temperature $\geq$ 38·0°C, report of fever in the past 48 hours, or other illness (will be referred to the nearest health facility for further evaluation)<br>— Reported pregnancy or breastfeeding in women who have had menarche but no menses in the past 4 weeks (assessed in women and girls ≥10 years of age)<br>— Children <9 months of age or <7 kg<br>— Known allergy or history of adverse reaction to DP<br>— Already taken 2 courses of DP in the past year or taken 1 course within the past 2 months<br>— Moderate or severe renal or hepatic insufficiency<br>— Currently with severe malaria<br>— Family history of sudden death or of congenital prolongation of the QTc interval.<br>— Known congenital prolongation of the QTc-interval or any clinical condition known to prolong the QTc interval.<br>— History of symptomatic cardiac arrhythmias or with clinically relevant bradycardia. Any predisposing cardiac conditions for arrhythmia such as severe hypertension, left ventricular hypertrophy (including hypertrophic cardiomyopathy) or congestive cardiac failure accompanied by reduced left ventricle ejection fraction.<br>— Electrolyte disturbances, particularly hypokalaemia, hypocalcaemia or hypomagnesaemia (including vomiting in child)<br>— Recent treatment with medicinal products known to prolong the QTc interval that may still be circulating at the time that DP is commenced (e.g. mefloquine, halofantrine, lumefantrine, chloroquine, quinine and other antimalarial agents)

Abbreviations: RACD, reactive case detection; rfMDA, reactive focal mass drug administration The Target Area is defined for rfMDA clusters as all individuals residing within 200m of an index case that is detected in passive surveillance and resides in a rfMDA study cluster, with individuals residing immediately beyond 200m included if a minimum of 30 individuals are not enrolled within 200m. The Target Area is defined for RACD clusters as all individuals residing within 500m of an index case that is detected in passive surveillance and resides in a RACD cluster.

Appendix 2. Characteristics of index cases and target population, mean % across clusters (95% CI) during two-year follow-up

Cluster level characteristic	Overall n=47*	RACD n=22**	rfMDA n=25***
INDEX CASES			
Male	48·5 (38·4 – 58·5)	48·5 (32·4 – 64·7)	48·5 (34·8 – 62·1)
Age (years)			
<15	15·3 (8·9 – 21·6)	15·8 (7·1 – 24·4)	14·8 (5·0 – 24·6)
15–40	60·2 (50·4 – 70·0)	59·7 (43·4 – 76·0)	60·6 (48·0 – 73·3)
>40	22·3 (13·4 – 31·3)	24·4 (8·6 – 40·1)	20·5 (9·8 – 31·3)
International travel in past 8 weeks	35·7 (25·6 – 45·9)	37·6 (20·5 – 54·7)	34·1 (21·0 – 47·2)
Occupation			
Agricultural	17·7 (8·7 – 26·6)	20.3 (6.4 – 34.2)	15.3 (2.9 – 27.8)
Manual labor	7·8 (2·3 – 13·2)	5.7 (0 – 11.7)	9.6 (0.55 – 18.6)
Manufacturing	2·4 (0 – 5·0)	3.1 (0 – 7.9)	1.8 (0 – 4.6)
Office	3·0 (0·22 – 5·8)	4.3 (0 – 9.7)	1.9 (0 – 4.6)
Small market sales/trade	5·4 (1·8 – 9·0)	5.5 (0 – 11.3)	5.3 (0.45 – 10.2)
Unemployed/Retiree	35·2 (24·1 – 46·4)	42.3 (23.7 – 60.9)	29.0 (15.0 – 43.0)
Student	21·7 (13·0 – 30·4)	13.9 (5.4 – 22.5)	28.6 (14.0 – 43.1)
Other	1·4 (0·19 – 2·6)	1.0 (0 – 2.9)	1.8 (0.05 – 3.5)
Child, non-student	3·2 (1·1 – 5·2)	3.6 (0.28 – 7.0)	2.7 (0.01 – 5.4)
Home sprayed in the past year			
Yes	17.7 (10.1 – 25.4)	5.3 (0 – 11.3)	28.6 (16.4 – 40.8)
No	71.8 (62.0 – 81.5)	82.2 (68.2 – 96.1)	62.6 (49.2 – 76.0)
Own bednet			
Yes	22.9 (13.9 – 31.9)	20.0 (8.1 – 31.8)	25.6 (11.5 – 39.6)
No	74.9 (65.3 – 84.4)	79.9 (68.0 – 91.8)	70.4 (55.3 – 85.6)
TARGET POPULATION			
Male	44.6 (39.4 – 49.7)	38.6 (31.8 – 45.4)	50.5 (43.2 – 57.8)
Age (years)			
<15	41.8 (37.1 – 46.6)	42.7 (34.5 – 49.8)	41.0 (34.2 – 47.8)
15–40	40.9 (36.1 – 45.7)	41.1 (33.0 – 49.2)	40.7 (34.6 – 46.7)
>40	17.3 (14.4 – 20.1)	16.2 (12.4 – 20.0)	18.3 (13.8 – 22.9)
International travel	2.8 (0.40 – 5.2)	2.3 (0.19 – 4.3)	3.3 (0 – 7.9)
Occupation			
Agricultural	15.2 (6.6 – 23.8)	7.8 (0 – 18.1)	22.6 (8.7 – 36.6)
Manual labor	3.0 (1.6 – 4.4)	3.5 (0.79 – 6.2)	2.4 (1.3 – 3.7)
Manufacturing	0.89 (0.39 – 1.4)	0.27 (0 – 0.66)	1.5 (0.63 – 2.4)
Office	1.7 (0.90 – 2.5)	1.7 (0.59 – 2.8)	1.7 (0.45 – 3.0)
Small market sales/trade	2.5 (1.4 – 3.5)	2.6 (0.96 – 4.3)	2.3 (0.99 – 3.5)
Unemployed/Retiree	27.9 (23.4 – 32.4)	33.1 (26.4 – 39.8)	22.7 (17.0 – 28.4)
Student	31.6 (26.0 – 37.2)	29.3 (21.5 – 37.1)	33.9 (25.3 – 42.5)
Other	2.7 (0 – 6.3)	4.1 (0 – 11.5)	1.2 (0 – 2.5)
Child, non-student	14.5 (11.5 – 17.6)	17.5 (12.3 – 22.7)	11.5 (8.4 – 14.6)
Household sprayed in past year (individual level)			
Yes	26.6 (16.3 – 36.9)	18.8 (5.4 – 32.2)	34.4 (18.4 – 50.4)
No	68.3 (57.4 – 79.2)	76.7 (63.0 – 90.3)	60.0 (42.5 – 77.4)
Don’t know	5.0 (1.8 – 8.3)	4.4 (0 – 9.0)	5.7 (0.61 – 10.8)
Household sprayed in past year (household level)			
Yes	25.2 (15.4 – 35.0)	21.6 (7.8 – 35.4)	28.5 (13.6 – 43.3)
No	69.2 (58.5 – 79.8)	72.9 (58.5 – 87.3)	65.7 (49.1 – 82.5)
Don’t know	5.6 (2.4 – 8.8)	5.5 (1.8 – 9.3)	5.7 (0.34 – 11.1)

Abbreviations: RACD, reactive case detection; rfMDA, reactive focal mass drug administration.

Data are mean proportion (95% CI). Some estimates with larger standard errors had lower bounds less than 0.
* n=39 for target population. N for target population is smaller than for index population because some index cases did not get an intervention response (index case coverage was not 100%).
** n=19 for target population. N for target population is smaller than for index population because some index cases did not get an intervention response (index case coverage was not 100%).
*** n=20 for target population. N for target population is smaller than for index population because some index cases did not get an intervention response (index case coverage was not 100%).

Vilakati S, *et al. BMJ Global Health* 2021; 6:e005021. doi: 10.1136/bmjgh-2021-005021

Appendix 3. Cluster-level incidence in the year prior to the trial (A) and during the trial (B) by intervention arm

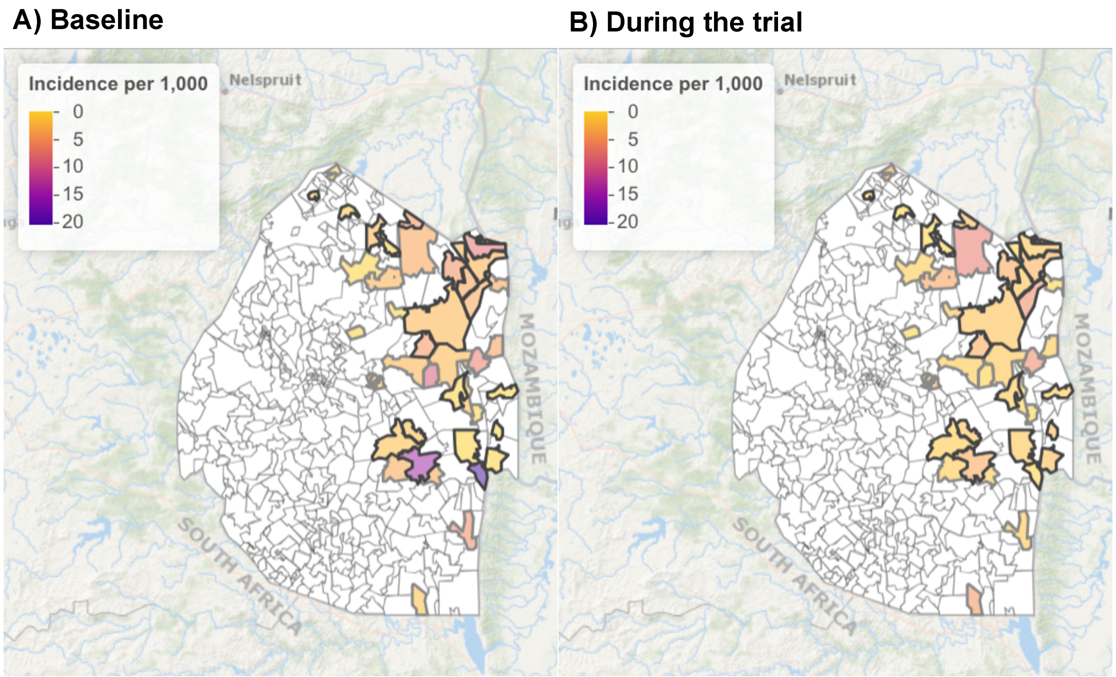

Map of the study area with malaria incidence per 1 000 by cluster. rfMDA clusters have black outlines and RACD clusters have grey outlines. Clusters not included in the trial are shown in white.

Vilakati S, *et al. BMJ Global Health* 2021; 6:e005021. doi: 10.1136/bmjgh-2021-005021

Appendix 4. Per-protocol analysis: Adjusted incidence rate ratios (IRRs) in 2015–2017 comparing clusters assigned to RACD versus rfMDA

Study Arm	N clusters	Incidence (cases per 1000 person-years)	Crude IRR	p-value	Adjusted IRR*	p-value
All cases						
RACD	19	2.10<br>(1.65 – 2.66)	1 (Ref)	0.94	1 (Ref)	0.98
rfMDA	18	2.22<br>(1.78 – 2.79)	1.03<br>(0.55 – 1.92)		0.99<br>(0.51 – 1.95)	
Local cases only						
RACD	19	0.97<br>(0.71 – 1.34)	1 (Ref)	0.99	1 (Ref)	0.52
rfMDA	18	1.34<br>(1.00 – 1.80)	1.00<br>(0.48 – 2.10)		0.73<br>(0.28 – 1.90)	

Abbreviations: RACD, reactive case detection; rfMDA, reactive focal mass drug administration
95% Confidence intervals for incidence were estimated using the Wilson method. Incidence rate ratios (IRRs) compared cluster-level incidence in the rfMDA arm to the RACD arm using an intention-to-treat approach and negative binomial models.
*Adjusted for baseline covariates that were associated with the outcome: incidence from July 2014–August 2015 (All cases model), local incidence from July 2014–August 2015 (Local cases only model).

Vilakati S, *et al. BMJ Global Health* 2021; 6:e005021. doi: 10.1136/bmjgh-2021-005021

Appendix 5. Cluster-level incidence in contiguous clusters by arm

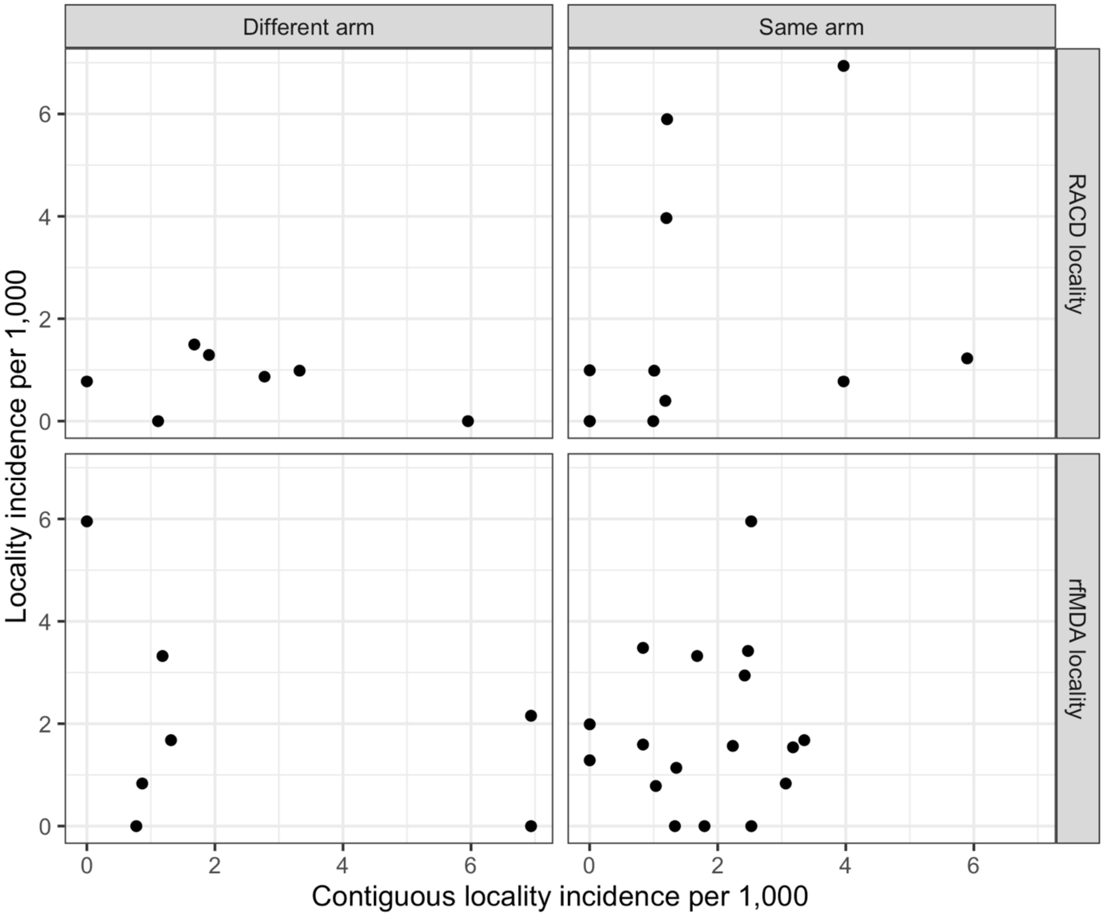

Abbreviations: RACD, reactive case detection; rfMDA, reactive focal mass drug administration. Due to the small number of clusters in each panel, we did not formally assess statistical correlations between the incidence in study localities and their contiguous neighboring localities.

Appendix 6. Symptomology of reported adverse events

Symptom	N=1,932* n (%)
Headache	37 (1.9)
Nausea and/or vomiting	25 (1.3)
Abdominal pain	17 (0.9)
Body weakness	11 (0.6)
Fever	7 (0.4)
Diarrhoea	7 (0.4)
Rash	5 (0.3)
Dizziness	4 (0.2)
Chest pain and/or breathing difficulty	3 (0.2)
Cough	2 (0.1)
Red, itchy eyes	2 (0.1)

*number of individuals who received dihydroartemisinin-piperaquine (DP)

Appendix 7. Symptomology in participants with reported adverse events that did not complete the entire course of dihydroartemisinin-piperaquine (n=5)

Individual No.	Age (years)	Sex	Symptoms	Suspected relationship to study drug	Decision to stop therapy (self, nurse)	Details of therapy discontinuation	Confirmed recovery	Time to recovery
1	33	Female	Vomiting	Possible	Nurse	Discontinued after vomitting with first and second doses.	Yes	1 day
2	27	Female	Nausea, fatigue	Probable	Self	Discontinued after first dose due to impact on activities of daily living, including ability to attend work.	Yes	2 days
3	49	Male	Difficulty breathing	Probable	Self	Discontinued after second dose when symptoms started.	Yes	A few hours
4	26	Female	Hyperventilation, difficulty breathing, chest tightening, chest pains, nausea, vomiting, stomach ache, diarrhoea	Definite	Nurse	Discontinued after second dose when nurse became aware of symptoms and participant newly disclosed history of hypertension. No hospitalisation was required.	Yes	Not recorded
5	13	Male	Nausea and vomiting	Possible	Self	Discontinued after nausea and vomiting with second dose.	Yes	A few hours
